# Feasibility of Point-of-Care Testing for Influenza Within a National Primary Care Sentinel Surveillance Network in England: Protocol for a Mixed Methods Study

**DOI:** 10.2196/14186

**Published:** 2019-11-11

**Authors:** Simon de Lusignan, Uy Hoang, Harshana Liyanage, Ivelina Yonova, Filipa Ferreira, Javier Diez-Domingo, Tristan Clark

**Affiliations:** 1 Nuffield Department of Primary Care Health Sciences University of Oxford Oxford United Kingdom; 2 Department of Clinical and Experimental Medicine University of Surrey Guildford United Kingdom; 3 Vaccine Research Department Foundation for the Promotion of Health and Biomedical Research in the Valencian Region Valencia Spain; 4 Academic Unit of Clinical and Experimental Sciences University of Southampton Southampton United Kingdom

**Keywords:** diagnosis, influenza, human, point-of-care systems, general practice

## Abstract

**Background:**

Point-of-care testing (POCT) for influenza promises to provide real-time information to influence clinical decision making and improve patient outcomes. Public Health England has published a toolkit to assist implementation of these tests in the UK National Health Service.

**Objective:**

A feasibility study will be undertaken to assess the implementation of influenza POCT in primary care as part of a sentinel surveillance network.

**Methods:**

We will conduct a mixed methods study to compare the sampling rates in practices using POCT and current virology swabbing practices not using POCT, and to understand the issues and barriers to implementation of influenza POCT in primary care workflows. The study will take place between March and May 2019. It will be nested in general practices that are part of the English national sentinel surveillance network run by the Royal College of General Practitioners Research and Surveillance Centre. The primary outcome is the number of valid influenza swabs taken and tested by the practices involved in the study using the new POCT.

**Results:**

A total of 6 practices were recruited, and data collection commenced on March 11, 2019. Moreover, 312 swab samples had been collected at the time of submission of the protocol, which was 32.5% (312/960) of the expected sample size. In addition, 68 samples were positive for influenza, which was 20.1% (68/338) of the expected sample size.

**Conclusions:**

To the best of our knowledge, this is the first time an evaluation study has been undertaken on POCT for influenza in general practice in the United Kingdom. This proposed study promises to shed light on the feasibility of implementation of POCT in primary care and on the views of practitioners about the use of influenza POCT in primary care, including its impact on primary care workflows.

**International Registered Report Identifier (IRRID):**

DERR1-10.2196/14186

## Introduction

### Background

Influenza is associated with high levels of morbidity and mortality [1]. Vaccination is suboptimally effective at preventing influenza in certain groups [2], and antivirals may improve clinical outcome, especially when administered early in the course of the disease [3].

The Royal College of General Practitioners’ (RCGP) Research and Surveillance Centre (RSC) program of influenza and respiratory disease surveillance has been established since 1967, making it the longest established primary care sentinel network in Europe [4,5]. Its work contributes to several important public health outputs including the early identification of pandemics and the assessment of vaccine effectiveness.

Current antiviral treatment for influenza needs to be administered within 48 hours from the onset of symptoms for optimal efficacy [6,7], although new antiviral agents have recently been developed, which may prove more effective. These new antivirals such as Baloxavir promise to improve time to resolution of symptoms and reduce complications for patients with influenza [8], although their use will likely be restricted to patients with microbiologically confirmed diagnosis, given their cost.

However, currently only a small proportion of patients with influenza-like illness (ILI) in primary care undergo microbiological testing, with few patients receiving antiviral medications appropriately according to guidelines [9].

### Point-of Care-Testing

Recently, highly accurate rapid molecular test platforms for influenza have become commercially available to hospitals and health care clinics. These near-patient or point-of-care tests (POCT) can produce results in under 30 minutes and so could be used to direct the use of antiviral medications for treatment and chemoprophylaxis of influenza [10,11]. Rapid molecular diagnostic testing for influenza has the potential to (1) improve clinical decision making regarding the use of antibiotics and antivirals, (2) improve patient outcomes due to the early appropriate use of antivirals, and (3) provide better information to inform sentinel surveillance and clinical research including studies of vaccine effectiveness and real-world trials.

In the United Kingdom, 2 commercially available, highly accurate molecular POCT platforms for influenza have recently been given the Conformité Européenne marking, the Cobas Liat test produced by Roche Diagnostics and the Abbott ID Now (formerly the Alere influenza A and B) test produced by Abbott Diagnostics [12].

Public Health England has advised that institutions interested in using POCT for influenza should consider a set of predefined questions (Multimedia Appendix 1) before implementation of these new tests [13]. However, these considerations do not specifically relate to implementation of POCT in primary care. In addition, guidelines have not been given regarding the specimen numbers needed for seasonal influenza situational awareness, which would be necessary for influenza disease surveillance in primary care [14].

### Objectives

We conducted this study to determine the feasibility of POCT for influenza in primary care, comparing its implementation with current practice for influenza specimen sampling within the RCGP RSC surveillance network, including the views of practitioners about the challenges of incorporating influenza POCT into primary care workflow.

## Methods

### Study Design

We will perform a mixed methods study to compare the sampling rates using POCT and current virology sampling practices within the RCGP RSC network and to understand the issues and barriers to the implementation of influenza POCT in primary care workflows.

### Study Setting and Population

The study will take place from March to May 2019. It will be completed at the end of the influenza season (when no cases will be detected during 2 consecutive weeks or equivalent).

The study will be nested in the English national sentinel surveillance network run by the RCGP RSC. Previous work has shown that the age and gender distribution of patients in the sentinel network is broadly similar to the English National census distribution, although there is a significantly higher proportion of both males and females in the 25 to 44 years age band, when compared with the census, and a lower proportion of people in the 0 to 4 years age band [4].

### Data Collection and Analysis

We will recruit 6 practices with a registered population of between 30,000 and 60,000 patients. Clinicians in the study practices will be encouraged to undertake nasal swabs from consented patients aged over 6 months presenting with an acute ILI (with symptoms of 5 days or less). The swabs will be tested for influenza in the practice using the POCT machine by the clinician or trained practitioner. The test will take approximately 15 min for a result to be displayed, and the clinician will be encouraged to record this result in the patients’ medical record.

Currently, virology sampling practices within the sentinel networks take up to a maximum of 20 samples per week per practice before (to look for circulating flu) and during (to see which strains are circulating and any drift) the season and when the season is over. Moreover, 35% (881/2591) of the specimens collected through the sentinel network are laboratory-confirmed influenza. Thus, over a 10-week study period, we would expect 1200 samples to be taken with 427 influenza positive swabs.

The primary outcome is the number of valid influenza swabs taken and tested by the practices in the RCGP RSC network using the new POCT.

The baseline characteristics of the patients who receive influenza testing with POCT will be compared with other patients who receive influenza specimen sampling within the sentinel surveillance network in terms of the following:

- Age group (6 months to 14 years, 15 to 64 years, and ≥65 years)
- Sex
- Chronic conditions that may make that individual more vulnerable to influenza infection (such as chronic pulmonary disease, cardiovascular disease, metabolic disorders, renal disease, treatment-induced immunosuppression and disease-induced immunosuppression, and medically attended obesity)
- Pregnancy status
- Socioeconomic status
- Ethnicity
- Influenza vaccination status or contraindication to influenza vaccination
- Pneumococcal vaccination
- Antibiotic and antiviral prescription
- Use of statins
- Smoking behavior or parental smoking behavior (for children)
- Perinatal and congenital risk factors (eg, birth weight and/or maturity at birth, perinatal factors, inborn errors of metabolism, and relevant malformations and congenital syndromes)
- Number of siblings (for children)
- Adherence to the local childhood vaccination program (for children)
- Number of health care visits 12 months before the study period, describing a study subjects’ health care seeking behavior

Number of hospitalizations 12 months before the study period is to be used as proxy for the severity of the chronic conditions.

The primary quantitative data collection will be undertaken at each practice. Data processing will be coordinated by researchers at the Department of Clinical and Experimental Medicine of the University of Surrey. Any data provided to the research team will be stored in their secure servers, which are compliant with the relevant legal and National Health Service digital information governance requirements. Aggregated data will be presented from the final analysis and will not contain any patient identifiable information.

Secondary outcomes will include the utility of the influenza POCT in primary care and the issues and barriers to implementation of influenza POCT in primary care workflows. Data for secondary outcomes will be collected by a semistructured survey of clinicians/practice staff at the participating practices (see Multimedia Appendix 2). Information will be collected about the following domains previously found to be important to implementation of POCT sampling:

- Performance of the POCT platform
- Clinical pathways and training
- Result reporting
- Clinical governance
- Costs
- Monitoring of effectiveness.

The semistructured survey and results reported will be used to assess which practices were more successful at integrating POCTs into their practice processes by comparing business process models of the practices. Business process models are graphical representations of business-oriented processes within an organization. This is helpful to model collaborations and business transactions within health systems. Business processes are typically modeled using the Business Process Modeling Notation (BPMN). BPMN can be used to depict the end-to-end flow of a business process. The notation has been specifically designed to coordinate the sequence of processes and the messages that flow between different process participants in a related set of business activities [15].

## Results

### Ethical Approval

The study was funded in July 2018 by the Development of Robust and Innovative Vaccine Effectiveness (DRIVE) European Union, Innovative Medicines Initiative project. The study received ethical approval from the UK Health Research Authority on February 4, 2019, Integrated Research Application System reference: 252081, Research Ethics Committee reference: 19/WM/0015. Moreover, 6 practices were recruited to the study, and data collection commenced on March 11, 2019.

### Initial Findings

In addition, 312 swab samples had been collected at the time of submission of the manuscript, which was 32.5% (312/960) of the expected sample size. Furthermore, 68 samples were positive for influenza, which was 20.1% (68/338) of the expected sample size. Qualitative interviews were being undertaken with practices, and the full results are expected to be published in summer 2019.

## Discussion

### Principal Findings

Our main finding from this study so far is that 312 influenza swabs have thus far been taken and tested using the new POCT machines in practices that are part of the RCGP RSC sentinel surveillance network.

To the best of our knowledge, this is the first time an evaluation study has been undertaken on POCT for influenza in general practice in the United Kingdom. Accurate, real-time monitoring of infectious diseases using rapid diagnostic POCT has been shown to improve outbreak preparedness and response compared with existing surveillance systems [16]. This proposed study will focus on the feasibility of implementation of POCT sampling in primary care versus current methods of influenza sampling in a sentinel network and on the views of practitioners about the use of influenza POCT in primary care, including its impact on primary care workflows.

The RCGP RSC sentinel network is appropriate for this research as the national sentinel surveillance network for England, and the implementation of POCT for influenza in general practices within this network have a potential to rapidly influence clinical care and public health surveillance.

### Limitations of the Study

#### Generalizability

Although the study will be conducted in primary care, the general practices involved in the RCGP RSC sentinel surveillance network may not be representative of general practices as a whole in the United Kingdom, as seen by the higher average practice scores in payment for results and higher average vaccination rates of practices within the network.

#### Sample Size

The small sample size used for this study makes it difficult to generalize our findings to a wider group of general practices.

## Appendix

Multimedia Appendix 1 Point-of-care testing for influenza—implementation checklist.

Multimedia Appendix 2 Semistructured questionnaire for primary care staff.

## Abbreviations

BPMN: Business Process Modeling Notation
DRIVE: Development of Robust and Innovative Vaccine Effectiveness
ILI: influenza-like illness
POCT: point-of-care testing
RCGP: Royal College of General Practitioners
RSC: Research and Surveillance Centre
