# Supplementary material for: Feasibility of Point-of-Care Testing for Influenza Within a National Primary Care Sentinel Surveillance Network in England: Protocol for a Mixed Methods Study
Source: JMIR Res Protoc. 2019 Nov 11;8(11):e14186. doi: 10.2196/14186 (PMC6878097; doi:10.2196/14186)
Supplement: Multimedia Appendix 1 [file resprot_v8i11e14186_app1.pdf]

Appendix 1 - Point of Care Test for Influenza – implementation checklist

Relevant implementation questions to consider from PHE[13]	Relevant implementation questions to consider for a sentinel surveillance network	Relevant implementation questions to consider for primary care practices
**Performance of the POCT platform**		
○ Which platform chosen<br>○ Rationale for choice<br>○ Location of platform<br>○ Test operator	○ Which platform chosen<br>○ Rationale for choice	○ Location of POCT<br>○ Test operator
**Clinical pathways and training**		
○ Clinical algorithm provided<br>○ Method used to disseminate algorithm<br>○ How will a test be ordered – including patient consent<br>○ Who will train staff to use POCT<br>○ How will they be assessed<br>○ Who is responsible for training and maintaining competency<br>○ Will you appoint a POCT team<br>○ When will the roll out of training begin	○ Who will train practices to use POCT<br>○ How will practices be assessed<br>○ Who is responsible for training and maintaining competency of practices<br>○ When will the roll out of training begin for practices	○ Clinical algorithm provided<br>○ Method used to disseminate algorithm to clinical staff<br>○ How will a test be ordered – including patient consent<br>○ Who will train practice staff to use POCT<br>○ How will practice staff be assessed<br>○ Who is responsible for training and maintaining competency<br>○ Will practices appoint a POCT team<br>○ When will the roll out of training begin for practice staff
**Result reporting**		
○ Where is result reported for real time action	○ Is data from POCT integrated into the sentinel surveillance	○ Where is result reported for real time clinical actions

○ Is it integrated into the medical record – if not how is the result available to clinicians<br>○ Does the result link to clinical protocols for management of flu<br>○ How are results flagged to the infection control team<br>○ How does this affect patient workflow in real time (isolation and cohorting)	system and available for public health analysis	○ Is it integrated into the medical record – if not how is the result available to clinicians<br>○ Does the result link to clinical protocols for management of flu<br>○ How are results flagged to the infection control team<br>○ How does this affect patient workflow in real time (isolation and cohorting)
**Clinical governance**		
○ Who is responsible for the POCT machine<br>○ Is there a clear line of accountability for any issue<br>○ Who is responsible for stock supply<br>○ Do you intent to do lab/ clinical verification of POCT results<br>○ Quality assurance considerations	○ Is there a lead contact for POCT within the sentinel surveillance network<br>○ Do you intent to do lab verification of POCT results<br>○ Quality assurance considerations	○ Who is responsible for the POCT machine<br>○ Is there a clear line of accountability for any issue<br>○ Who is responsible for stock supply<br>○ Do you intent to do lab/ clinical verification of POCT results<br>○ Quality assurance considerations
**Costs**		
○ Estimated number of test over the winter period<br>○ Estimate cost per test, initial costs for the platform<br>○ Estimated savings	○ Estimated number of test over the winter period<br>○ Estimate cost per test, initial costs for the platform	○ Estimated savings
**Monitoring of effectiveness**		

○ Components to be assessed (eg length of stay, proportion of NAI, treated patients with or without flu, proportion of flu positive patients given inappropriate antibiotics)?<br>○ Where will the information be stored?<br>○ When will this be reviewed?<br>○ Who will be responsible for this?	○ When will information about effectiveness be reviewed?<br>○ Who will be responsible for this?	○ Components to be assessed (eg length of stay, proportion of NAI, treated patients with or without flu, proportion of flu positive patients given inappropriate antibiotics)?<br>○ Where will the information be stored?<br>○ When will this be reviewed?<br>○ Who will be responsible for this?
