# Supplementary material for: Feasibility of Point-of-Care Testing for Influenza Within a National Primary Care Sentinel Surveillance Network in England: Protocol for a Mixed Methods Study
Source: JMIR Res Protoc. 2019 Nov 11;8(11):e14186. doi: 10.2196/14186 (PMC6878097; doi:10.2196/14186)
Supplement: Multimedia Appendix 2 [file resprot_v8i11e14186_app2.pdf]

Appendix 2 – Semi-structured questionnaire for primary care staff

- Performance of the POCT platform

  - Location of POCT
  - Who was the POCT operator

- Clinical pathways and training

  - Was a clinical algorithm created that included the POCT?
  - Method used to disseminate algorithm to practice staff?
  - How was a test ordered (including how was patient consent obtained)?
  - Who trained practice staff to use POCT?
  - How were practice staff assessed?
  - Who was responsible for training and maintaining competency of practice staff?
  - Did practices appoint a POCT team?
  - When will the roll out of training begin for practice staff?

- Result reporting

  - Where were POCT result reported for real time clinical actions?
  - Were the POCT results integrated into the medical record – if not how were the result available to clinicians?
  - Did the POCT result link to clinical protocols for management of flu?
  - How were POCT results flagged to the infection control team?
  - How did access to POCT results affect patient workflow in real time (isolation and cohorting)?

- Clinical governance

  - Who was responsible for the POCT machine?
  - Was there a clear line of accountability for any issues with the POCT?
  - Who was responsible for stock supply?
  - Was there any intent to do clinical verification of the POCT result?
  - Was any quality assurance of POCT machines undertaken?

- Costs

  - Estimated savings

- Monitoring of effectiveness

  - Were any of the following patient outcomes monitored (eg length of stay, proportion of NAI, treated patients with or without flu, proportion of flu positive patients given inappropriate antibiotics)?
  - Where was this information stored?
  - When was this reviewed?
  - Who was responsible for this monitoring?
